# Microneedle–Tissue Interaction Across Varying Biological and Mechanical Conditions

**DOI:** 10.3390/bios15080521

**Published:** 2025-08-09

**Authors:** Elham Lori Zoudani, Prabuddha De Saram, Kyle Engel, Nam-Trung Nguyen, Navid Kashaninejad

**Affiliations:** 1 Queensland Quantum and Advanced Technologies Research Institute, Griffith University, 170 Kessels Road, Nathan, QLD 4111, Australia; 2 Centre for Regional and Rural Futures (CeRRF), Centre for Sustainable Bioproducts (CSB), School of Engineering, Deakin University, Geelong, VIC 3216, Australia; 3 School of Engineering and Built Environment, Griffith University, 170 Kessels Road, Nathan, QLD 4111, Australia

**Keywords:** microneedles, tissue adhesion, drug delivery, biosensing, insertion force, extraction force

## Abstract

Microneedle (MN)–tissue interactions play a critical role in the efficiency and reliability of transdermal drug delivery and biosensing, yet their mechanistic understanding remains limited. This study systematically investigates the effects of biological (tissue type and temperature) and mechanical (needle design, material, and insertion velocity) parameters on the performance of microneedle insertion and extraction. Experiments were performed on porcine skin, chicken breast, and agarose gel to represent varying tissue properties. Additionally, the effect of tissue temperature on replicating physiological conditions, such as hypo- and hyperthermia, was evaluated using porcine skin as the sample. A novel conical MN design integrated with surface suction-cup structures was developed to improve tissue adhesion. Mechanical responses were analyzed through force–displacement measurements, evaluating insertion force, extraction force, and relaxation time. Results show that elevated tissue temperature reduces insertion and extraction forces while shortening relaxation times, indicating increased tissue compliance. The suction-cup MNs significantly enhanced needle–tissue adhesion, with the most pronounced effect observed in chicken breast tissue, achieving more than a four-fold increase in extraction force compared to conventional conical needles. These findings provide valuable insights into optimizing the design of MNs for advanced biomedical applications.

## 1. Introduction

Microneedles (MNs) are a promising class of transdermal delivery systems, offering numerous advantages over traditional methods [1]. Over three decades of research on MN-based drug delivery [2,3,4] and sensing [5,6,7] systems have demonstrated their potential to overcome challenges associated with conventional delivery techniques. Beyond their ease of administration, MNs provide additional benefits due to their unique interaction with the epidermis, which serves as the delivery pathway. The epidermis is rich in antigen-presenting cells (APCs), facilitating enhanced immune responses in applications such as vaccine delivery [8,9]. Moreover, MNs have shown significant promise in targeted and controlled drug release applications as well as in wearable biosensor technologies [10,11].

The development of MNs must adhere to specific criteria that align with their primary objectives: convenience and efficacy. Successful MN penetration serves as the foundation for practical MN applications, a characteristic that is consistent across various applications ranging from drug delivery to biosensing. One of the most critical yet often overlooked aspects is the needle–tissue interaction, which spans the entire process from initial insertion to full penetration and eventual extraction of the MN array. The needle–tissue interaction is a critical determinant of the system’s overall performance. This knowledge enables the optimization of needle design for specific applications. A thorough understanding of this interaction leads to improved clinical outcomes, enhanced patient comfort, and reduced tissue damage.

Microneedles experience a series of forces applied to the needle shaft during the insertion and removal procedures, i.e., insertion force and extraction force [12,13]. These forces must be maintained within a controlled range to minimize any potential issues arising from excessive force values. The insertion force refers to the force required for MNs to penetrate tissue. It should be minimized to reduce pain during application [14]. However, it must not be so low that it results in partial insertion, which could compromise the overall effectiveness of the process [15]. Extraction force refers to the force required to remove MNs after they have been used. This force should be sufficient to maintain reliable adhesion, particularly in applications that demand sustained contact with tissue [16,17,18], but must not exceed a threshold that compromises the convenience of the application [19].

The human body consists of various tissue types, each with distinct inherent properties, i.e., hydration, elasticity, and stiffness. Additionally, these tissue properties can change under both normal and extreme physiological conditions. These variations may influence the performance of MN insertion. Understanding these factors is essential for advancing personalized medicine, as the design of MNs and insertion conditions can be tailored to meet an individual’s specific medical needs.

In the ongoing effort to optimize MN performance, numerous studies, both simulation-based and experimental, have explored enhancements in MN morphology [18,20,21,22,23,24] and material properties [25,26], primarily in the context of medical applications such as drug delivery. A deeper understanding of the fundamental mechanics of the needle–tissue penetration process is essential for bridging the gap between device design and functional performance across diverse biological environments.

This study aims to present a different aspect of MN research, focusing on factors that influence needle–tissue interaction, with the primary objective of providing a framework for optimizing MNs. The insertion and extraction performance of a three-dimensional (3D) printed MN array was experimentally analyzed. While much of the existing MN research aimed at performance optimization has predominantly focused on the geometry and structural design of the microneedles themselves, limited attention has been paid to the characteristics of the target tissue. The primary objective of the present study is to emphasize the importance of a more holistic approach, considering not only microneedle design factors but also the mechanical and structural properties of the tissue. To this aim, two main categories of influencing parameters were identified: internal factors, related to the properties of the target tissue (e.g., tissue type and temperature), and external factors, associated with the MN’s structural characteristics and insertion conditions.

**Figure 1:**
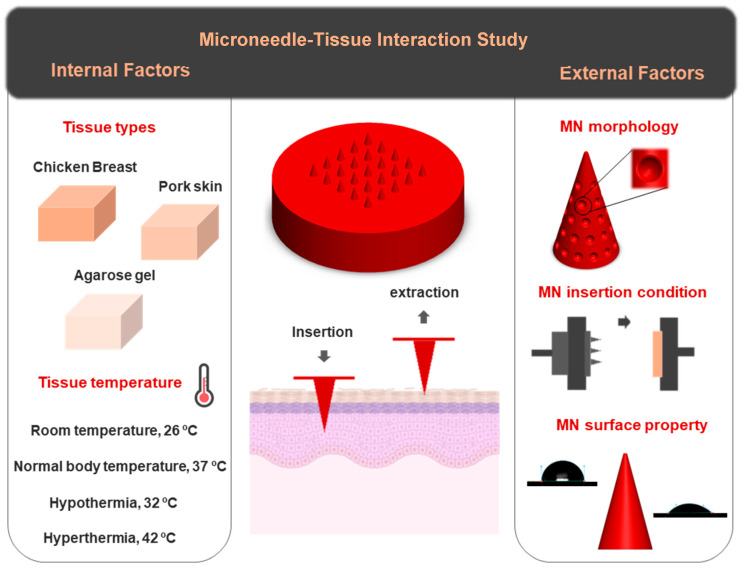
Schematic illustration of the experimental parameters investigated in this study, highlighting two major categories of factors influencing MN–tissue interactions: internal factors (tissue type and temperature) and external factors (MN morphology, insertion conditions, and surface properties). Tissue types include chicken breast, porcine skin, and agarose gel. Different temperatures (26 °C, 32 °C, 37 °C, and 42 °C) were tested for porcine skin to simulate physiological variations. Some elements (tissue model and thermometer) were created with BioRender. https://BioRender.com/.

Figure 1 provides a schematic overview of the key parameters investigated in this study. Different tissue types, including chicken breast, pork skin, and agarose gel, have been selected as the tissue models, each resembling a specific body part. The effect of tissue temperature on needle–tissue interaction was analyzed across a range of physiologically relevant temperatures: room temperature (26 °C), body temperature (37 °C), hypothermia (32 °C), and hyperthermia conditions (42 °C). As one of the external factors, a new shape of MN (a cone consisting of suction cups on the structure) has been proposed for tissue adhesion application. The effects of MN surface wettability and insertion velocity were also examined as other external influencing parameters. The aim of this study is to systematically assess the influence of each parameter on MN–tissue interaction, thereby informing future design strategies for more efficient and application-specific MN platforms.

## 2. Materials and Methods

Materials: Tissue samples, chicken breast, and pork skin were purchased from a local butchery. Agarose (BioReagent, for molecular biology; low EEO, Sigma-Aldrich, St. Louis, MO, USA) was used for the preparation of the non-fibrous tissue model. The tissue and MN patch holders were made of Poly (methyl methacrylate) (PMMA).

Equipment: A Boston microfabrication (BMF) S230 high-resolution digital light projection (DLP) 3D printer (Boston Micro Fabrication (BMF), Maynard, MA, USA) was used for the fabrication of MNs. The surface morphology of the microneedles was analyzed using a scanning electron microscope (SEM) (Apreo 2S, Thermo Scientific, Hillsboro, OR, USA). Low vacuum mode was selected for imaging, with a voltage of 20 kV and an amperage of 0.1 nA. The wettability of the patch material substrate was evaluated through water contact angle measurement using an optical tensiometer (Theta Flex, Biolin Scientific, Finland). A custom-built delaminator (DTS Company, 705 Wallea Drive, Menlo Park, CA, USA) was used for MN insertion/extraction analysis. Plasma cleaner (PDC-002-CE, Harrick Plasma, Ithaca, NY, USA) was used to treat the simple conical MN patch and modify the surface chemistry of the needles. An ultrasonic cleaner (Model C1040) was used to regulate pork tissue temperature during experiments for tissue temperature effect analysis.

MN fabrication: Additive manufacturing of the devices was performed using a BMF S230 high-resolution DLD 3D printer. The BMF BIO resin used in this process was supplied by Embedded Logic Solutions Pty Ltd., Sydney, NSW, Australia. Photopolymerization was conducted using the S230’s top-down projection system, operating at a maximum power density of 218.2 mW/cm^2^, through a 50 µm-thick taut Fluorinated Ethylene Propylene (FEP) film. CAD 3D models were prepared using Voxeldance Additive Slicing software that allowed the modification of the layer thickness mid-print. This feature was used to optimize print times by adjusting layer thicknesses as needed to enhance surface feature resolution. The BMF S230 utilizes a ceramic roller to ensure the layer is printed to the programmed thickness. This is accomplished by rolling the upper surface of the FEP membrane prior to the projection of light for each layer. However, to prevent any possible damage to the small surface features and any damage to the FEP membrane, rolling was disabled for layers containing surface features.

During all 3D printing procedures, adequate dwell times according to BMF specifications were used to allow for the settling of turbulent resin and enhance layer accuracy.

Post-printing procedures included careful washing of all parts in filtered (Millipore, 0.45 µm) Propan-2-ol for 30 min, with solvent replacement. Once no resin residue remained, the parts were allowed to air dry prior to post-print curing. Curing and annealing were carried out using a Formlabs Form Cure for 10 min, 50 °C, and 405 nm exposure.

Tissue sample preparation: A 2% agarose gel was prepared by dissolving 2 g of agarose powder in 100 mL of distilled water. First, the agarose powder was poured into a beaker, followed by the addition of the measured distilled water. The mixture was then heated in a microwave for 2 min until the powder was fully dissolved, resulting in a clear solution. After allowing the solution to cool, it was poured into a transparent polystyrene cube-shaped container for solidification. Pork skin and chicken breast were stored at 4 °C in a refrigerator immediately after purchase and used within 24 h. Prior to testing, the tissues were removed from the fridge and allowed to thaw to room temperature, ensuring thermal equilibrium. The agarose gel, chicken breast, and pork skin were then trimmed into uniform pieces with dimensions of 3 cm × 3 cm.

Pork tissue preparation for temperature effect analysis: Pork skin tissue samples were prepared at defined temperatures to assess the influence of tissue temperature on MN performance. The tissue was placed in a glass beaker filled with water and secured using a holder to prevent contact with the bottom surface. The beaker was then immersed in an ultrasonic cleaner, which was used solely as a temperature-controlled water bath. Importantly, the ultrasonic function was not activated during this process. The bath was set to the target temperature for each trial (26 °C, 32 °C, 37 °C, or 42 °C). The ultrasonic cleaner’s bath was uniformly heated to ensure consistent temperature distribution and to minimize the risk of tissue damage. The tissue remained in the chamber for 15 min to allow for temperature stabilization prior to testing.

MN insertion/extraction test: The tissue samples were mounted onto the moving stage of the delaminator setup by attaching them to a custom-fabricated poly(methyl methacrylate) (PMMA) holder. The MN patch was secured to the opposing stage, which was equipped with a load cell, using double-sided tape for a stable attachment to its corresponding PMMA holder. During testing, the tissue stage advanced toward the MN patch at a constant speed of 100 µm/s. After full insertion and completion of the relaxation period, the stage reversed direction, retracting the tissue at the same velocity to perform MN withdrawal.

Each test was repeated three times, and the results are reported as the mean ± standard deviation (SD).

## 3. Results and Discussion

### 3.1. Design Validation and Surface Morphology of MN

**Figure 2:**
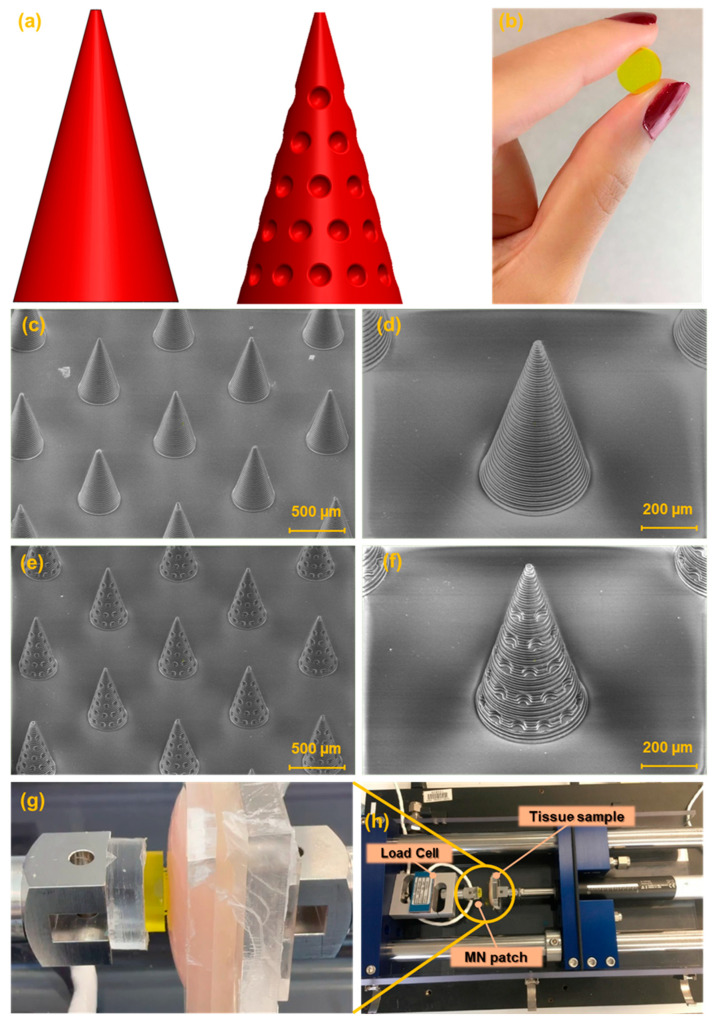
(**a**) Schematic of the CAD design of the simple conical MN and the new MN design (cone integrated with suction cup-like structures). (**b**) Image of 3D-printed MN patch. SEM images of (**c**,**d**) simple conical MN array. (**e**,**f**) New shape of MNs (cone integrated with suction cups). (**g**,**h**) image of the delaminator (setup of the experiment).

Two MN designs were developed for this study: a conventional smooth conical shape and a modified conical shape featuring regularly spaced hemispherical cavities intended to enhance tissue adhesion, Figure 2a. Figure 2b presents the image of the fabricated MN array. The SEM images of the fabricated MNs are shown in Figure 2c,d. The general shape of the needles was successfully printed in a cone shape with base circle radius of 200 µm. Needle tips look sharp with a radius of 20 µm. The height of the MNs was chosen as 700 µm. The array contains 25 (5 × 5) needles in a square distribution pattern with needle interspacing of 1000 µm.

### 3.2. Microneedle Insertion–Extraction Test

The mechanical performance of the MNs was evaluated using a custom-built platform based on a delaminator device, designed for force–displacement characterization (see Figure 2g,h). In the experimental setup, the tissue sample was securely affixed to the moving stage to prevent any undesired movement during testing. In contrast, the MN patch was mounted on a fixed stage equipped with a calibrated load cell to ensure accurate force measurements.

The moving stage was set to advance toward the fixed stage at a controlled speed of 100 µm/s, gradually bringing the tissue into contact with the needle tips. Upon initial contact, the system was reset, then the stage continued to move forward to allow full MN insertion into the tissue. Throughout this process, force–displacement data were collected using computer software, beginning from the moment of initial contact until complete needle insertion. The load cell recorded both loading and unloading forces, while the displacement was monitored via the position of the stage.

**Figure 3:**
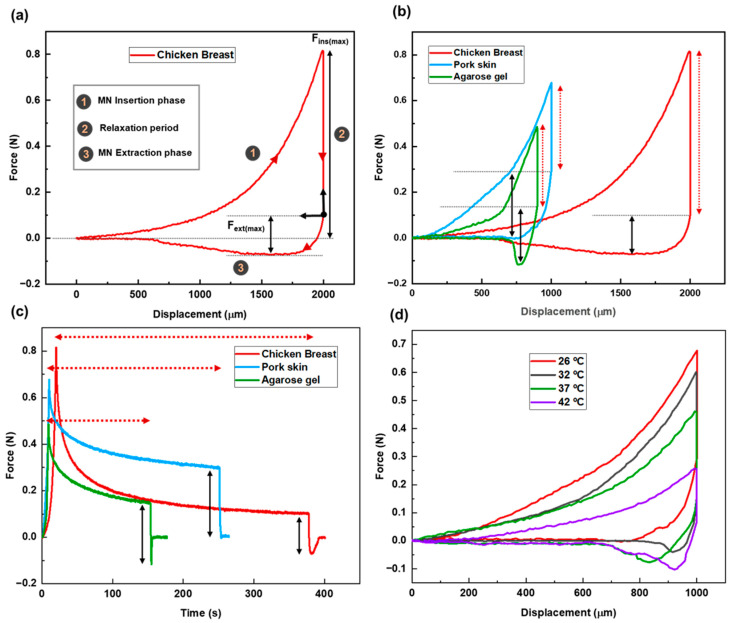
(**a**) Force–displacement graph for MN insertion/extraction test into chicken breast as the tissue model. (**b**) Force vs. displacement graph for MN insertion/extraction test for three different tissues (chicken breast, pork skin, agarose gel) as representative of different human body parts. Red dashed arrows and solid black arrows represent relaxation period and maximum extraction force, respectively. (**c**) Force vs. time graph for different tissues. Red dashed arrows and solid black arrows represent relaxation period and maximum extraction force, respectively. (**d**) Force–displacement graph for the pork skin as the tissue sample at different temperatures (26 °C, 32 °C, 37 °C, 42 °C) simulating different physiological conditions.

Figure 3a presents the force–displacement curve for MN penetration and extraction using chicken breast as the tissue model. The zero point on the graph represents the position where the needle tips first touch the tissue. The total needle displacement was set to 2000 µm, which corresponds to approximately 2.8 times the needle height, ensuring full penetration and accounting for the elastic deformation of the tissue.

The stages of MN insertion and withdrawal are indicated on the graph. Stage 1 specifies the insertion stage. As the MN patch advances into the tissue, the applied force increases, reflecting the disruption of the tissue matrix and the need to overcome the mechanical resistance of internal structures. The maximum insertion force, measured once the MNs have fully penetrated the tissue, is recorded at 813 ± 4 mN.

After full insertion, the needles were left embedded in the tissue for a period defined as the relaxation or dwelling time (Stage 2). This phase allows for the assessment of the tissue’s viscoelastic behavior, its time-dependent mechanical response to sustained deformation. Viscoelasticity encompasses two primary components: elastic behavior, representing the tissue’s ability to recover instantaneously upon load removal, and viscous behavior, which reflects a gradual, time-dependent deformation under constant stress.

During this phase, the force gradually decreases from its peak value and eventually stabilizes and reaches an equilibrium. This relaxation allows the tissue to conform around the MNs and adhere to their surfaces. The duration of this period tightly connects to the tissue properties. The recorded relaxation period for this case was 358 ± 5 s.

Following the relaxation period, the extraction phase begins (Stage 3). According to the force–displacement graph, the pull-out process starts from the equilibrium state after relaxation. This point is set as the origin of the extraction phase. The extraction force is calculated by subtracting the equilibrium force from the recorded force values during the extraction phase. As the patch is withdrawn, static friction between the tissue and MN surfaces causes a gradual increase in the extraction force. As seen in the graph, the slope is steeper at the initial stage, reaching the lowest point on the curve. The maximum extraction force required for MN detachment is 172 ± 5 mN. After the peak extraction force is reached, the force decreases as the MNs slide out of the tissue, eventually approaching zero.

Overall, the force–displacement curve effectively illustrates the mechanical interaction between the MNs and the tissue throughout the entire insertion–dwell–extraction cycle. This behavior is influenced by several factors, including internal parameters such as tissue composition, structure, and temperature, as well as external parameters such as MN geometry, material properties, and insertion velocity.

The following sections will provide a detailed discussion on how each of these factors influences MN–tissue interaction dynamics.

#### 3.2.1. Internal (Tissue-Related) Factors

##### Comparative Analysis of MN Insertion–Extraction in Different Tissues

The MN insertion–extraction tests were conducted on three distinct tissue models—agarose gel, chicken breast, and pork skin—with measured Young’s moduli of 150 ± 5 kPa, 82 ± 8 kPa, and 425 ± 6 kPa, respectively. These models are widely recognized and commonly employed in microneedle-related research due to their representative mechanical properties [22,27]. The corresponding force–displacement curves for each model are presented in Figure 3b. Each tissue model was selected to mimic different types of human tissue. Agarose gel represents soft, hydrated tissues such as the human eye or wound sites; chicken breast simulates soft muscle tissue; and pork skin serves as an analog for stiffer, more fibrous skin tissue.

The insertion and extraction behavior of the MNs varied significantly across these tissue types, as reflected in key mechanical parameters, including insertion force, the required needle displacement for full insertion, relaxation time, and extraction force.

Based on their structural characteristics, the tissues were classified into two groups of fibrous tissues, chicken breast and pork skin, and non-fibrous tissue, agarose gel.

These structural differences were clearly visible in the force–displacement profiles (Figure 3b), with each tissue exhibiting a unique mechanical response to MN penetration and withdrawal. To ensure full MNs penetration, displacement distances were tailored to each tissue’s mechanical properties: chicken breast: ~2000 µm displacement; pork skin: ~1000 µm displacement; agarose gel: ~900 µm displacement. These values were selected to guarantee full needle penetration. Complete penetration was identified by a noticeable inflection point in the force–displacement curve, corresponding to a sudden increase in force, which indicated contact between the MN base and the tissue surface.

During the insertion stage, the slope of the force–displacement curve for pork skin is the steepest, followed by agarose gel and then chicken breast. This indicates that the rate of force increase is highest for pork skin, which reflects its greater stiffness compared to other tested tissues.

Among the three tissue types, chicken breast exhibited the longest relaxation time (358 ± 5 s), indicating a strong viscous component and slower adaptation to MN insertion. Pork skin showed a moderate relaxation time (241 ± 3 s). Agarose gel had the shortest relaxation time (144 ± 6 s), suggesting a more elastic structure with a faster mechanical recovery following deformation. Figure 3c presents the force vs. time graph for the three cases, clearly illustrating the duration of the relaxation period (red dashed arrows).

The overall force–displacement trends during the withdrawal process reveal an abrupt force transition in the agarose gel curve, implying a sudden structural failure and rapid response upon MN removal. In contrast, pork skin and chicken breast exhibit more gradual force changes during extraction. This behavior is likely attributed to the more elastic nature of agarose gel, in contrast to the viscoelastic characteristics of the biological tissues.

The relatively stiff nature of pork skin leads to a higher extraction force during MN removal compared to the softer tissues of agarose gel and chicken breast. The maximum extraction forces recorded for pork skin, chicken breast, and agarose gel are 298 ± 3, 172 ± 5, and 234 ± 4 mN, respectively.

Table 1 presents the data regarding MN insertion and extraction processes for the three tested tissue samples.

##### Tissue Temperature

In this section, we investigate the effect of tissue temperature on the mechanical interaction between MNs and tissue. Pork skin was chosen as the tissue model. A range of temperatures was applied to simulate different physiological conditions. Specifically, four temperature levels were tested: 26 °C (room temperature), 32 °C (hypothermia), 37 °C (normal body temperature), and 42 °C (hyperthermia condition).

The force–displacement curves reveal that both insertion and extraction forces decrease as tissue temperature increases (see Figure 3d). This trend is attributed to enhanced molecular mobility at higher temperatures, which causes the tissue to soften and expand. As a result, MNs can more easily penetrate and withdraw from the tissue, requiring less force. The maximum insertion forces recorded were 664 ± 2 mN, 602 ± 2 mN, 458 ± 5 mN, and 258 ± 4 mN at 26 °C, 32 °C, 37 °C, and 42 °C, respectively. Correspondingly, the maximum extraction forces were 298 ± 3 mN, 198 ± 2 mN, 184 ± 3 mN, and 171 ± 4 mN for the same temperature conditions.

Temperature also influenced the relaxation time following MN insertion. Tissues at higher temperatures exhibited shorter relaxation periods. Since relaxation time serves as an indicator of the viscoelastic properties of tissue, and viscoelasticity is inversely related to temperature, this behavior suggests that warmer tissues adapt more quickly to the presence of MNs. Consequently, they display more elastic-like behavior under thermal conditions. Table 2 presents the data regarding MN insertion and extraction processes for the pork tissue at different temperatures.

#### 3.2.2. External Factors

In addition to the elements related to the nature of the tissue, there are other factors that require careful consideration, as they directly impact the needle–tissue interaction. These factors are primarily associated with external conditions, such as those related to the MN itself and the conditions governing the insertion process of the MN.

##### Microneedle Physical Morphology

As one of the primary factors that directly affect the needle–tissue interaction, the physical morphology of MNs significantly influences their insertion and extraction performance. This is especially evident in tissue adhesion applications, where the MN’s ability to firmly anchor to tissue is essential. Among the various methods proposed so far as MN–tissue adhesion enhancers, morphological modifications have gained significant interest. Specific structural features incorporated into MN designs have been shown to enhance the extraction force [22], an essential metric for evaluating MN–tissue adhesion.

Building upon this concept, inspired by the suction cup structures found in octopi’s arms, biological features known for their strong adhesion, we developed a novel MN patch featuring conical MNs integrated with suction cup-like structures. This new design, illustrated in Figure 2a, followed by its SEM images in Figure 2e,f, features a conical body integrated with 40 suction cups distributed across its surface. In addition to improved adhesiveness, this configuration offers a larger surface area compared to the simple conical counterpart—533,354 µm^2^ versus 483,650 µm^2^. The increased surface area also facilitates greater drug loading capacity, potentially enhancing drug delivery efficiency.

**Figure 4:**
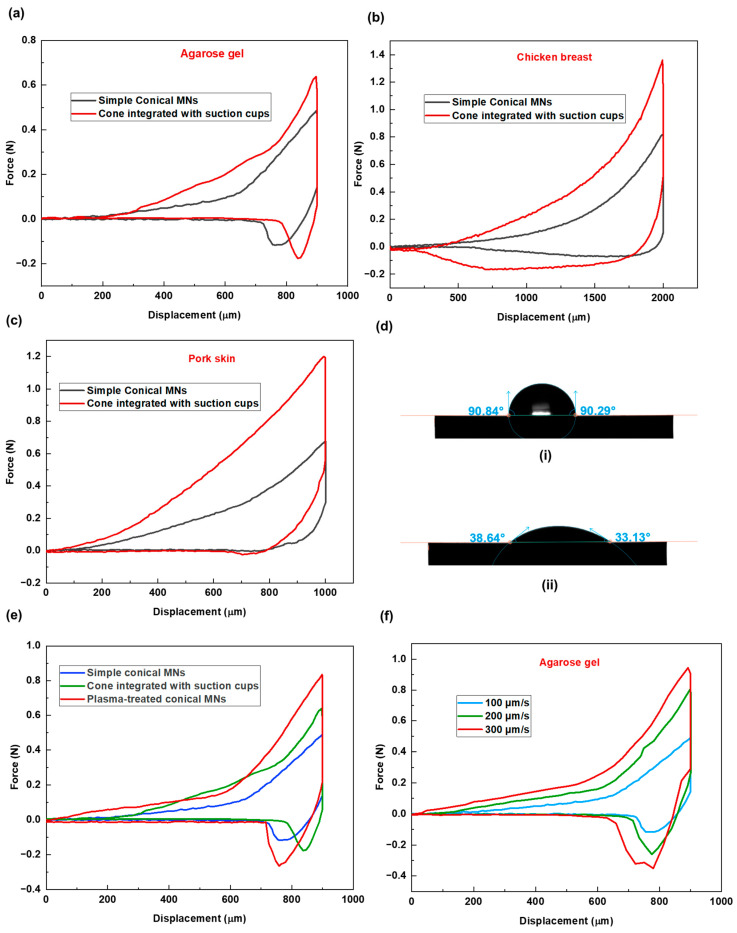
Force–displacement graphs for two MN shapes across three different tissues of (**a**) agarose gel, (**b**) chicken breast, and (**c**) pork skin. (**d**) Water contact angle measurement on (**i**) non-treated (**ii**) plasma-treated material substrate. (**e**) Force vs. displacement graphs of simple conical, cone integrated with cup-like structures, and plasma-treated MNs, demonstrating the effect of MN surface treatment on needle–tissue mechanical behavior; the test was performed for agarose gel as the tissue sample. (**f**) Force vs. displacement graph demonstrating the effect of MN insertion velocity on needle–tissue mechanical behavior; the test was performed for agarose gel as the tissue sample.

To evaluate the performance of this new design, comparative experiments were conducted using three tissue models: agarose gel, chicken breast, and pork skin. The corresponding force–displacement graphs are presented in Figure 4a–c. Across all tissue types, MNs with suction cups demonstrated higher insertion forces, indicating increased resistance during penetration.

Following insertion, tissues interacting with the suction cup design consistently displayed more extended relaxation periods, which is due to the additional time required for the tissue to accommodate the structural indentations.

Subsequently, higher extraction forces were recorded for the new design across all samples, confirming enhanced adhesion as a result of increased surface contact and the interaction between the tissue and the microneedles’ surface features. Specifically, the tissue penetrates and becomes entrapped within the concave suction cup structures, which resemble small bowls. These structures promote tissue interlocking and result in increased adhesion. A closer analysis of the force–displacement data reveals varying levels of adhesion response across tissue types. The relative increases in insertion and extraction forces between the simple and suction cup designs were as follows: agarose gel—insertion force increased by 1.4×, extraction force by 1.134×; pork skin—insertion force increased by 1.768×, extraction force by 2.01×; chicken breast—insertion force increased by 1.6×, extraction force by 4.03×. These differences can be attributed to the inherent structural properties of each tissue. The soft, sticky, and compliant nature of chicken breast allows it to conform more easily to the concave suction cup structures, resulting in greater tissue interlocking and higher adhesive response.

The new MN design performed most effectively in chicken breast tissue, where a relatively modest increase in insertion force was accompanied by a substantial gain in extraction force. This suggests that the effectiveness of MN morphology in promoting tissue adhesion may be tissue-dependent. Therefore, MN design should be tailored to the specific properties of the target tissue, rather than adopting a one-size-fits-all approach. Table 3 presents data regarding MN insertion and extraction processes for the new MN design for the three tested tissue samples.

##### Microneedle Surface Chemical Properties

Continuing our discussion on external factors influencing the behavior of MNs within tissue, we now focus on the chemical composition of MNs.

To modify the surface chemistry of the microneedles (MNs), plasma treatment was applied. Contact angle measurement (Figure 4d) on the material substrate confirmed a significant increase in hydrophilicity following 15 s plasma treatment under low-pressure mode.

Following this treatment, MN penetration tests were conducted using agarose gel as the tissue model. A 2% agarose gel (prepared with 2 g of agarose in 100 mL of water), known for its high water content, was selected to evaluate the impact of increased microneedles’ surface hydrophilicity on insertion/extraction performance. As shown in the force versus displacement graph (Figure 4e), there is a noticeable difference in the insertion and extraction phases for the treated MNs compared to the non-treated sample. The results demonstrated a further increase in tissue adhesion (higher extraction force), surpassing even the suction cup design. The maximum extraction forces were 481 ± 2, 294 ± 4, and 234 ± 4 mN for the treated, suction-cup, and non-treated simple conical MNs, respectively. This enhanced adhesion is likely due to increased affinity between the MN surface and the agarose gel with a high water content. Furthermore, the comparatively longer relaxation period (264 ± 4 s) observed for the treated MNs is attributed to persistent MN–tissue surface molecular interactions.

These findings further highlight the importance of considering tissue composition when designing MNs. For example, in tissues with higher water content, such as wound tissues, this option of providing MNs with a hydrophilic surface wetting property could function synergistically with the morphological modification aspect to further improve the system’s efficacy.

Table 4 presents the data regarding MN insertion and extraction processes for the treated MNs, the new MN design, and the non-treated sample.

##### Insertion Velocity

As an external factor influencing how MNs interact with tissue during insertion and extraction, the conditions under which MNs are inserted are also important. To this aim, three different insertion velocities of 100, 200, and 300 µm/s have been considered for the penetration study of solid conical MN patch in agarose gel. The graph of the MN insertion–extraction study shows differences in force–displacement values for these three cases (see Figure 4f). In that, higher insertion and extraction forces have been recorded for conditions with higher insertion velocity. This is because, at higher insertion velocities, the tissue becomes stiffer and more resistant to MN penetration due to insufficient time for relaxation. Similarly, the abrupt deformation caused by rapid insertion results in the needles becoming more tightly embedded in the tissue, thereby increasing the extraction force.

Table 5 presents the data regarding MN insertion and extraction processes for MN at different insertion velocities for the agarose gel tissue sample.

## 4. Conclusions and Limitations

In this study, we investigated the factors influencing MN–tissue interactions, categorizing them into two main groups: internal factors, related to the tissue properties, and external factors, associated with MN design and insertion conditions. An MN patch was fabricated from bioresin material for this purpose. To evaluate internal factors, various tissues, i.e., pork skin, chicken breast, and agarose gel, were selected, each representing a different analog of human tissue. We also explored the effect of tissue temperature on MN performance by exposing the pork skin tissue sample to various conditions: room temperature (26 °C), 32 °C (hypothermia simulation), 37 °C (normal body temperature), and 42 °C (hyperthermia simulation).

For the external factors, we introduced a novel MN design with a conical shape featuring suction cup-like structures on its surface. The behavior of this new design was examined across the different tissue types. Additionally, we assessed the influence of MN surface wettability on tissue interaction and evaluated the effect of varying insertion velocities on penetration performance. Overall, our findings demonstrate that both tissue-specific properties and MN design characteristics significantly influence device behavior and can be optimized for clinical studies on MN-based platforms.

In the field of microneedle applications, a significant research gap remains unaddressed. This gap primarily arises from the difficulty of working with real tissue samples, as access to human-like tissue analogs, closely resembling specific organs or body parts, is limited. Consequently, many studies have shifted toward using synthetic or artificial tissue models to evaluate microneedle functionality. However, it is important to acknowledge that these artificial models have inherent limitations and cannot fully replicate the complexity of in vivo tissues. Most notably, they lack circulatory systems and exhibit structural differences that limit their physiological relevance.

This challenge highlights a critical need for companies active in this field to invest in the development of advanced tissue models that more accurately mimic the mechanical and biological properties of real human tissues. As demonstrated in this study, microneedle performance is highly dependent on the tissue type with which it interacts. Therefore, such progress is essential for generating reliable and application-specific data. Furthermore, considering the high cost of microneedle production, having access to more precise and biologically relevant data is key to reducing development inefficiencies.

## Figures and Tables

**Table 1:** MN insertion/extraction key information for different tissue types. Each test was repeated three times, and the data are reported as mean ± SD.

Tissue Type	MN Displacement (µm)	Max Insertion Force (mN)	Relaxation Period (s)	Max Extraction Force (mN)
Chicken breast	2000	813 ± 4	358 ± 5	172 ± 5
Pork skin	1000	664 ± 2	241 ± 3	298 ± 3
Agarose gel	900	471 ± 4	144 ± 6	234 ± 4

**Table 2:** MN insertion/extraction key information for tissues with different temperatures. Each test was repeated three times, and the data are reported as mean ± SD (standard deviation).

Tissue Temperature	Max Insertion Force (mN)	Relaxation Period (s)	Max Extraction Force (mN)
Room temperature (26 °C)	664 ± 2	241 ± 4	298 ± 3
Hypothermia (32 °C)	602 ± 2	225 ± 2	198 ± 2
Normal body temperature (37 °C)	458 ± 5	212 ± 4	184 ± 3
Hyperthermia (42 °C)	258 ± 4	186 ± 2	171 ± 4

**Table 3:** Microneedle insertion/extraction key characteristics for the new shape of the MNs cross different tissues. Each test was repeated three times, and the data are reported as mean ± SD.

Tissue Type	MN Displacement (µm)	Max Insertion Force (mN)	Relaxation Period (s)	Max Extraction Force (mN)
Chicken breast	2000	1334 ± 3	405 ± 2	689 ± 3
Pork skin	1000	1200 ± 5	363 ± 4	598 ± 3
Agarose gel	900	680 ± 5	194 ± 5	294 ± 4

**Table 4:** MN insertion/extraction key characteristics for the non-treated conical MNs, new shape, and plasma-treated MN patch in agarose gel as the tissue model. Each test was repeated three times, and the data are reported as mean ± SD.

MN Type	MN Displacement (µm)	Max Insertion Force (mN)	Relaxation Period (s)	Max Extraction Force (mN)
Simple cone shape	900	471 ± 4	144 ± 6	234 ± 4
Cone integrated with suction cups	900	680 ± 5	194 ± 5	294 ± 4
Plasma-treated MNs	900	816 ± 4	264 ± 4	481 ± 2

**Table 5:** MN insertion/extraction key characteristics for different insertion velocities for agarose gel tissue sample. Each test was repeated three times, and the data are reported as mean ± SD.

Insertion Velocity	MN Displacement (µm)	Max Insertion Force (mN)	Relaxation Period (s)	Max Extraction Force (mN)
100 µm/s	900	471 ± 4	144 ± 6	234 ± 4
200 µm/s	900	779 ± 5	175 ± 3	527 ± 3
300 µm/s	900	943 ± 2	190 ± 4	641 ± 5

## Data Availability

The data that support the findings of this study are available from the corresponding author upon reasonable request.
